# The “Super-Fontan” Phenotype: Characterizing Factors Associated With High Physical Performance

**DOI:** 10.3389/fcvm.2021.764273

**Published:** 2021-12-07

**Authors:** Derek L. Tran, David S. Celermajer, Julian Ayer, Leeanne Grigg, Carley Clendenning, Tim Hornung, Robert Justo, Glen M. Davis, Yves d'Udekem, Rachael Cordina

**Affiliations:** ^1^Department of Cardiology, Royal Prince Alfred Hospital, Sydney, NSW, Australia; ^2^Central Clinical School, The University of Sydney School of Medicine, Sydney, NSW, Australia; ^3^Charles Perkins Centre, Heart Research Institute, Sydney, NSW, Australia; ^4^Heart Centre for Children, The Sydney Children's Hospital Network, Sydney, NSW, Australia; ^5^The Children's Hospital at Westmead Clinical School, Sydney, NSW, Australia; ^6^Department of Cardiology, The Royal Melbourne Hospital, Melbourne, VIC, Australia; ^7^The University of Melbourne School of Medicine, Melbourne, VIC, Australia; ^8^Murdoch Children's Research Institute, Melbourne, VIC, Australia; ^9^Green Lane Paediatric and Congenital Cardiac Service, Starship Hospital, Auckland, New Zealand; ^10^Paediatric Cardiac Service, Queensland Children's Hospital, Brisbane, QLD, Australia; ^11^The University of Queensland School of Medicine, Brisbane, QLD, Australia; ^12^Discipline of Exercise and Sports Science, Sydney School of Health Sciences, Faculty of Medicine and Health, The University of Sydney, Sydney, NSW, Australia; ^13^The George Washington University School of Medicine and Health Sciences, Washington, DC, United States; ^14^Division of Cardiovascular Surgery, Children's National Hospital, Washington, DC, United States

**Keywords:** physical activity, congenital heart disease, exercise training, cardiac rehabilitation, exercise capacity

## Abstract

**Background:** People with a Fontan circulation usually have moderately impaired exercise performance, although a subset have high physical performance (“Super-Fontan”), which may represent a low-risk phenotype.

**Methods:** People with a “Super-Fontan” phenotype were defined as achieving normal exercise performance [≥80% predicted peak oxygen uptake (VO_2_) and work rate] during cardiopulmonary exercise testing (CPET) and were identified from the Australian and New Zealand Fontan Registry. A Fontan control group that included people with impaired exercise performance (<80% predicted VO_2_ or work rate) was also identified based on a 1:3 allocation ratio. A subset of participants were prospectively recruited and completed a series of physical activity, exercise self-efficacy, and health-related quality of life questionnaires.

**Results:** Sixty CPETs (“Super-Fontan”, *n* = 15; control, *n* = 45) were included. A subset (“Super-Fontan”, *n* = 10; control, *n* = 13) completed a series of questionnaires. Average age was 29 ± 8 years; 48% were males. Exercise capacity reflected by percent predicted VO_2_ was 67 ± 17% in the entire cohort. Compared to the “Super-Fontan” phenotype, age at Fontan completion was higher in controls (4.0 ± 2.9 vs. 7.2 ± 5.3 years, *p* = 0.002). Only one (7%) person in the “Super-Fontan” group had a dominant right ventricle compared to 15 (33%) controls (*p* = 0.043). None of those in the “Super-Fontan” group were obese, while almost a quarter (22%) of controls were obese based on body mass index (*p* = 0.046). Lung function abnormalities were less prevalent in the “Super-Fontan” group (20 vs. 70%, *p* = 0.006). Exercise self-efficacy was greater in the “Super-Fontan” group (34.2 ± 3.6 vs. 27.9 ± 7.2, *p* = 0.02). Self-reported sports participation and physical activity levels during childhood and early adulthood were higher in the “Super-Fontan” group (*p* < 0.05). The total average time spent participating in structured sports and physical activity was 4.3 ± 2.6 h/wk in the “Super-Fontan” group compared to 2.0 ± 3.0 h/wk in controls, *p* = 0.003. There were no differences in self-reported current total physical activity score or health-related quality of life between groups (*p* ≥ 0.05).

**Conclusions:** The “Super-Fontan” phenotype is associated with a healthy weight, lower age at Fontan completion, better exercise self-efficacy, and higher overall levels of sport and physical activity participation during physical development.

## Introduction

Francis Fontan first described the Fontan procedure in 1971 as a surgical method to treat babies born with tricuspid atresia (1). The procedure involves redirecting venous return directly into the pulmonary arteries resulting in no subpulmonary pump. The Fontan procedure has evolved with the advancement of medicine and surgical techniques in an attempt to optimize long-term outcomes, and although clinical outcomes have improved significantly, rates of morbidity and premature death are still high.

Exercise intolerance is common in people living with a Fontan circulation. Peak oxygen uptake (VO_2_) is the primary index of exercise tolerance (i.e., exercise capacity) and has significant prognostic value in patients with congenital heart disease (2). People with a Fontan circulation have reduced percent predicted peak VO_2_, which on average ranges from 60 to 65% (3, 4). However, there is extensive variability between patients, and it is acknowledged that a subgroup—“Super-Fontans”—have superior exercise performance (exercise and work capacity) compared to the majority of the Fontan population (5).

Currently, there is limited information about this subset of people who have superior physical performance. Since we originally described this unique phenotype (5), other centers have also characterized a similar subset of Fontan patients with normal exercise capacity (6, 7). Importantly, higher exercise capacity in people with a Fontan circulation appears to be associated with better prognosis and end-organ function (7–9). Understanding the factors associated with normal exercise capacity in this unique subset of patients can potentially aid in risk stratification and the identification of therapeutic targets. The aim of this study was to characterize factors associated with superior exercise performance in people with the “Super-Fontan” phenotype.

## Methods

People in the Australian and New Zealand Fontan Registry with recorded cardiopulmonary exercise testing (CPET) results and a “Super-Fontan” phenotype were identified and included in this study. People with impaired exercise capacity were also identified as controls based on a 1:3 allocation. Exercise and work capacity was measured by peak VO_2_ and work rate, respectively. To account for sex, height, and weight differences, peak VO_2_ and work rate are expressed as a percentage of predicted normal values (10, 11). Participants were categorized into a “Super-Fontan” (5) or a control group. The “Super-Fontan” group was defined as achieving normal exercise and work capacity (≥80% predicted) (5, 6, 12–15). The control group consisted of Fontan subjects who had reduced exercise or work capacity (<80% predicted).

We decided to include work capacity as a criterion as obese patients may have normal exercise capacity but present with limited work capacity and exercise intolerance. Participants were excluded if their CPET was conducted on a treadmill or if results were considered to be submaximal effort defined as a peak respiratory exchange ratio <1.0 (16). A subset of participants (study group) completed a series of health-related quality of life, physical activity, and exercise self-efficacy questionnaires. Clinical and demographic information, including dominant ventricular morphology, type of Fontan procedure, patent fenestration, sex, and age at Fontan completion, were obtained from the Australian and New Zealand Fontan Registry database and medical records when available. This study was approved by the Royal Children's Hospital Melbourne Human Research Ethics Committee (38,172).

### Exercise Self-Efficacy and Quality of Life

Exercise self-efficacy was assessed using the Exercise Self-Efficacy Scale (17). The total score was calculated as the sum of all questions, with a higher score reflecting greater exercise self-efficacy, which assesses an individual's beliefs in their ability to continue exercising regularly.

Health-related quality of life was measured using the PedsQL Adult Quality of Life Inventory Version 4. The items of each question were reversed scored, and linearly transformed in accordance with the scoring guidelines. In addition to the total score, the physical health summary score and psychosocial health summary score were also calculated, with higher scores suggesting better health-related quality of life.

### Cardiopulmonary Exercise Testing and Spirometry

Center specific CPET protocols were performed on an electronically braked cycle ergometer as part of routine clinical care. In addition to measures of peak VO_2_, work rate, and pre-exercise spirometry, CPET parameters including minute ventilation (VE), carbon dioxide production (VCO_2_), blood pressure, VO_2_ at the anaerobic threshold, heart rate (HR), and arterial oxygen saturation indicated by pulse oximetry were obtained when available. Predicted maximal oxygen pulse (ml/beat)—a surrogate for stroke volume—was calculated by dividing predicted maximal VO_2_ by predicted maximal HR (220–age) (18). A cardiovascular limitation to exercise performance was indicated by a chronotropic index (cardiovascular index) above the upper limit of normal (19) or a reduced peak oxygen pulse (<80% predicted). Chronotropic index was calculated as ΔHR (beats/min)/ΔVO_2_ (L/min) (19, 20). Maximal voluntary ventilation (MVV) was estimated as forced expiratory volume in 1 s (FEV_1_) x 40, and breathing reserve was calculated as MVV–peak VE or (MVV–peak VE)/MVV x 100. A mechanical ventilatory limitation to exercise was suggested by a breathing reserve of <15% or <11 L/min (18, 21). Peak circulatory power was calculated as peak VO_2_ (mL/kg/min) x peak systolic blood pressure (mm Hg). Ventilatory inefficiency was suggested by a peak VE/VCO_2_ ratio of >40. A significant fall in oxygen saturation was considered as a decrease of ≥5%.

Spirometry parameters were considered as abnormal if values were below the lower limit of normal calculated from the Global Lung Initiative regression equations (22). Lung function was defined as normal, obstruction, restriction, or mixed defect in accordance with the American Thoracic Society/European Respiratory Society algorithm (23). In the absence of total lung capacity measured by plethysmography, ventilatory restriction was suggested if forced vital capacity (FVC) was below the lower limit of normal. Mixed defect was suggested if the FEV_1_/FVC ratio and FVC were below the lower limit of normal.

### Physical Activity Across the Lifespan

To assess structured sport and physical activity participation across the lifespan, we used a modified version of the Kriska long-term recall physical activity questionnaire (24). Participants were asked to recall the sports and physical activities they participated in across multiple age ranges. For each sport and physical activity reported, the years in each age range, duration (hours) per month, and months per year were recorded. Sports and physical activities were categorized as childhood (ages 4–12 years), high school and early adulthood (ages 13–21 years), older adulthood (ages 22+ years), and physical activity across the lifespan (four to the age at questionnaire completion). The total hours of sport or physical activity participation were summated for each category and indexed as an average per week (h/wk).

### Current Level of Physical Activity

Self-reported current levels of physical activity and sedentary time were assessed using the International Physical Activity Questionnaire (IPAQ) Long-Form. The IPAQ scores were not truncated, and metabolic minutes per week (MET-min/week) were calculated in accordance with the IPAQ Scoring Manual. Sedentary activity was reflected by sitting time (minutes) per day.

### Statistical Analysis

Statistical analysis was performed using IBM SPSS version 26 software (IBM Corp, Armonk, NY, USA). Data are presented as mean ± standard deviation or number (%) unless specified otherwise. The Shapiro-Wilk test or visual inspection of histograms and Q-Q plots were conducted to assess for normal distribution. An independent *t*-test or Mann–Whitney U was used as appropriate to compare differences between the “Super-Fontan” group and the control group. Proportions were compared using Pearson Chi-Square. A *p*-value of < 0.05 was considered as statistically significant.

## Results

### Participant Demographics

Detailed participant demographics and characteristics are shown in Table 1. Of the 60 people with a Fontan circulation included in the CPET analysis, 15 had a “Super-Fontan” phenotype, and 35 had impaired exercise performance. The average age was 28.7 ± 7.6 years, and 48% were males.

**Table 1:** Participant demographics.

	**All Fontan Participants**		**“Super-Fontan”**		**Control**		***p*-value**
	***n***		***n***		***n***		
Sex (males), *n* (%)	60	29 (48.3%)	15	5 (33.3%)	45	24 (53.3%)	0.18
Age, years	60	28.7 ± 7.6	15	27.9 ± 5.7	45	28.9 ± 8.2	0.53
BMI, kg/m^2^	60	25.9 ± 4.7	15	24.4 ± 2.7	45	26.3 ± 5.1	0.34
Obese, *n* (%)	60	10 (16.7%)	15	0 (0%)	45	10 (22.2%)	**0.046**
**Type of Fontan**, ***n*** **(%)**	60		15		45		0.75^a^
APC		18 (30.0%)		4 (26.7%)		14 (31.1%)	
LT		23 (38.3%)		8 (53.3%)		15 (33.3%)	
ECC		19 (31.7%)		3 (20.0%)		16 (35.6%)	
**Dominant ventricle**, ***n*** **(%)**	60		15		45		**0.043** ^b^
Left		37 (61.7%)		13 (86.7%)		24 (53.3%)	
Biventricular		3 (5%)		1 (6.7%)		2 (4.4%)	
Indeterminant		4 (6.7%)		0 (0%)		4 (8.9%)	
Right		16 (26.7%)		1 (6.7%)		15 (33.3%)	
Age at Fontan palliation, years	60	6.4 ± 5.0	15	4.0 ± 2.9	45	7.2 ± 5.3	**0.002**
Patent fenestration, *n* (%)	60	10 (16.7%)	15	1 (6.7%)	45	9 (20%)	0.23
Time since Fontan palliation, years	60	22.2 ± 5.6	15	23.9 ± 4.2	45	21.7 ± 6.0	0.19

*APC, atriopulmonary connection; BMI, body mass index; ECC, extra cardiac conduit; LT, lateral tunnel*.

a *APC vs. total cavopulmonary connection*.

b *Dominant left ventricle, biventricular, or indeterminant ventricle vs. dominant right ventricle. Bold values denote statistical significance (p < 0.05)*.

The average body mass index (BMI) was 25.9 kg/m^2^, and 20 people (33%) were overweight or obese. None who had the “Super-Fontan” phenotype were obese based on BMI compared to 22% in the control group (*p* = 0.046). The majority (70%) had a total cavopulmonary connection, and the average age at Fontan procedure was 6.4 ± 5.0 years. The age at Fontan procedure was lower in the “Super-Fontan” group compared to the control group (4.0 ± 2.9 vs. 7.2 ± 5.3 years, *p* = 0.002). Thirty-seven (62%) had a dominant left ventricle, 16 (27%) had a dominant right ventricle, 3 (5%) had biventricular morphology, and 4 (7%) had indeterminate ventricles. A dominant right ventricle was associated with impaired exercise performance (*p* = 0.043). One person (7%) in the “Super-Fontan” group had a Fontan conversion from an atriopulmonary connection to an extracardiac conduit type circulation. There were no statistically significant differences in age, sex, BMI, type of Fontan procedure, or patent fenestration between groups (*p* ≥ 0.05 for all).

Twenty-three Fontan participants completed the questionnaires. Of the 23 Fontan study group participants, 10 (43%) were in the “Super-Fontan” group, and 13 (57%) were in the control group. There were no differences between groups in baseline demographics for the subset of study participants who completed the questionnaires (*p* ≥ 0.05 for all).

### Exercise Self-Efficacy and Health-Related Quality of Life

The average total exercise self-efficacy score was higher in the “Super-Fontan” group compared to the control group (34.2 ± 3.6 vs. 27.9 ± 7.2, *p* = 0.02).

There was no statistically significant difference in total health-related quality of life score between the “Super-Fontan” group and the control group (78.9 ± 13.0 vs. 68.2 ± 18.8, *p* = 0.14). There was also no statistically significant difference between the “Super-Fontan” and control groups in the physical health summary score (80.3 ± 11.7 vs. 67.1 ± 21.9, *p* = 0.1) or psychosocial health summary score (78.2 ± 15.4 vs. 68.8 ± 19.6, *p* = 0.23).

### Cardiopulmonary Exercise Testing and Spirometry

Detailed CPET and spirometry results are shown in Tables 2, 3. In the study group, the average time from the CPET to the questionnaires was 2.1 ± 1.9 years, and there was no difference between groups (*p* = 0.5).

**Table 2:** Lung function and cardiopulmonary exercise testing results.

	**All Fontan Participants**		**“Super-Fontan”**		**Control**		***p*-value**
	***n***		***n***		***n***		
FEV_1_, percent predicted	37	80.6 ± 11.7	10	85.7 ± 10.4	27	78.7 ± 11.7	0.11
FVC, percent predicted	37	79.9 ± 9.8	10	85.0 ± 7.8	27	78.0 ± 9.9	0.05
FEV_1_/FVC ratio	37	0.85 ± 0.06	10	0.85 ± 0.06	27	0.85 ± 0.06	0.93
Peak VO_2_, percent predicted	60	66.8 ± 17.1	15	89.4 ± 7.1	45	59.3 ± 12.0	**<0.001**
Peak work rate, percent predicted	60	71.7 ± 21.5	15	98.0 ± 9.7	45	63.0 ± 16.6	**<0.001**
VO_2_ at AT, percentage of predicted VO_2_	58	44.4 ± 14.9	14	60.2 ± 16.2	44	39.4 ± 10.3	**<0.001**
Oxygen pulse, percent predicted	60	87.0 ± 23.0	15	108.7 ± 15.6	45	79.7 ± 20.4	**<0.001**
Maximal HR, percent predicted	60	78.1 ± 13.7	15	83.3 ± 9.2	45	76.4 ± 14.6	0.09
HRR, bpm	60	65.7 ± 27	15	83.2 ± 20.4	45	59.9 ± 26.6	**0.003**
Chronotropic index, percent predicted	40	109.2 ± 36.9	11	99.1 ± 23.1	29	113.0 ± 40.6	0.19
Peak SpO_2_, percent	56	90.4 ± 7.2	13	91.2 ± 6.9	43	90.2 ± 7.4	0.57
ΔSpO_2_, percent	56	3.6 ± 4.6	13	4.3 ± 5.2	43	3.4 ± 4.5	0.56
Peak RER	60	1.21 ± 0.11	15	1.12 ± 0.07	45	1.22 ± 0.11	**0.04**
Peak circulatory power, mm Hg × mL/kg/min	53	3,425 ± 1,454	13	4,739 ± 1,519	40	2,998 ± 1,161	**<0.001**
Peak VE, L/min	60	72.1 ± 21.8	15	85.6 ± 21.7	45	67.7 ± 20.1	**0.01**
Peak VE/VCO_2_	60	37.3 ± 8.6	15	35.9 ± 8.3	45	37.7 ± 8.7	0.54
Breathing reserve, percent	37	35.5 ± 17.8	10	28.6 ± 23.6	27	38.1 ± 14.8	0.15

*AT, anerobic threshold; FEV_1_, forced expiratory volume in 1 s; FVC, forced vital capacity; HR, heart rate; HRR, heart rate reserve; RER, respiratory exchange ratio; SpO_2_, arterial oxygen saturation measured by pulse oximetry; VE, minute ventilation; VO_2_, oxygen uptake; VCO_2_, carbon dioxide production. Bold values denote statistical significance (p < 0.05)*.

**Table 3:** Lung function and cardiopulmonary exercise testing result categories.

	**All Fontan Participants**		**“Super-Fontan”**		**Control**		***p*-value**
	***n***		***n***		***n***		
**Lung function**, ***n*** **(%)**	37		10		27		**0.006** ^**a**^
Normal		16 (43.2%)		8 (80%)		8 (29.6%)	
Restriction		20 (54.1%)		2 (20%)		18 (66.7%)	
Obstruction		0 (0%)		0 (0%)		0 (0%)	
Mixed defect		1 (2.7%)		0 (0%)		1 (3.7%)	
Mechanical ventilatory limitation (yes), *n* (%)	37	5 (13.5%)	10	2 (20%)	27	3 (11.1%)	0.48
**Peak VE/VCO**_**2**_ **ratio**, ***n*** **(%)**	60		15		45		0.53
≤ 40		40 (66.7%)		11 (73.3%)		29 (64.4%)	
>40		20 (33.3%)		4 (26.7%)		16 (35.6%)	
**Chronotropic index**, ***n*** **(%)**	40		11		29		0.073^b^
Low		3 (7.5%)		0 (0%)		3 (10.3%)	
Low-normal		3 (7.5%)		0 (0%)		3 (10.3%)	
Normal		22 (55%)		10 (90.9%)		12 (41.4%)	
High-normal		8 (20%)		1 (9.1%)		7 (24.1%)	
High		4 (10%)		0 (0%)		4 (13.8%)	
**Oxygen pulse**, ***n*** **(%)**	60		15		45		**<0.001**
≥80% predicted		32 (53.3%)		15 (100%)		17 (37.8%)	
<80% predicted		28 (46.7%)		0 (0%)		28 (62.2%)	
Cardiovascular limitation (yes), *n* (%)	60	28 (46.7%)	15	0 (0%)	45	28 (62.2%)	**<0.001**
ΔSpO_2_ >5% (yes), *n* (%)	56	17 (30.4%)	13	5 (38.5%)	43	12 (27.9%)	0.47

*SpO_2_, arterial oxygen saturation measured by pulse oximetry; VCO_2_, carbon dioxide production; VE, minute ventilation*.

a *Normal lung function vs. restriction, obstruction or mixed defect*.

b *Normal range chronotropic index vs. low or high chronotropic index. A mechanical ventilatory limitation to exercise performance was suggested by a breathing reserve of <15% or <11 L/min. A cardiovascular limitation to exercise performance was suggested by a peak oxygen pulse <80% of predicted or a high chronotropic index. Bold values denote statistical significance (p < 0.05)*.

The average percent predicted peak VO_2_ and work rate for the entire cohort was 67 ± 17% and 72 ± 22%, respectively. There was no statistically significant difference in percent predicted maximum HR between the “Super-Fontan” and control groups (83 ± 9% vs. 76 ± 15%, *p* = 0.09). Peak circulatory power, HR reserve (HRR), peak VE, and VO_2_ at anaerobic threshold (percentage of predicted VO_2_) were higher in the “Super-Fontan” group (*p* < 0.05 for all). There was no difference in exercise-induced desaturation between groups (*p* = 0.5).

Of the 40 participants (“Super-Fontan,” *n* = 11; control, *n* = 29) where the chronotropic index could be calculated, 7 (18%) participants had values outside the normal range. All participants in the “Super-Fontan” group had a chronotropic index within the normal range. In the control group, 4 (14%) participants had a high chronotropic index, and 3 (10%) had a low chronotropic index suggesting cardiovascular limitation and chronotropic insufficiency as inhibitors to exercise performance, respectively. The “Super-Fontan” group also had a higher percent predicted oxygen pulse compared to the control group (109 ± 16% vs. 80 ± 20%, *p* < 0.001). When markers of cardiovascular limitation were combined [low peak oxygen pulse (<80% predicted) or a high chronotropic index], no patient with the “Super-Fontan” phenotype had evidence of a cardiovascular limitation to exercise capacity compared to 62% in the control group (*p* < 0.001).

Thirty-seven people with a Fontan circulation (“Super-Fontan,” *n* = 10; control, *n* = 27) had baseline spirometry recorded, 16 (43%) had normal spirometry function, 20 (54%) had evidence of ventilatory restriction, and 1 (3%) had a pattern suggestive of mixed defect. The “Super-Fontan” group tended to have higher percent predicted FVC compared to the control group (85 ± 8% vs. 78 ± 10%, *p* = 0.05). Lung function abnormalities at rest were associated with impaired exercise performance; 2 (20%) patients in the “Super-Fontan” group had ventilatory or mixed defect compared to 19 (70%) in the control group (*p* = 0.006).

The average breathing reserve was 36 ± 18%, and five out of the 37 patients had a mechanical ventilatory limitation to exercise performance. The majority (80%) who had a mechanical ventilatory limitation also had evidence of ventilatory restriction at rest.

### Physical Activity Across the Lifespan

**Figure 1:**
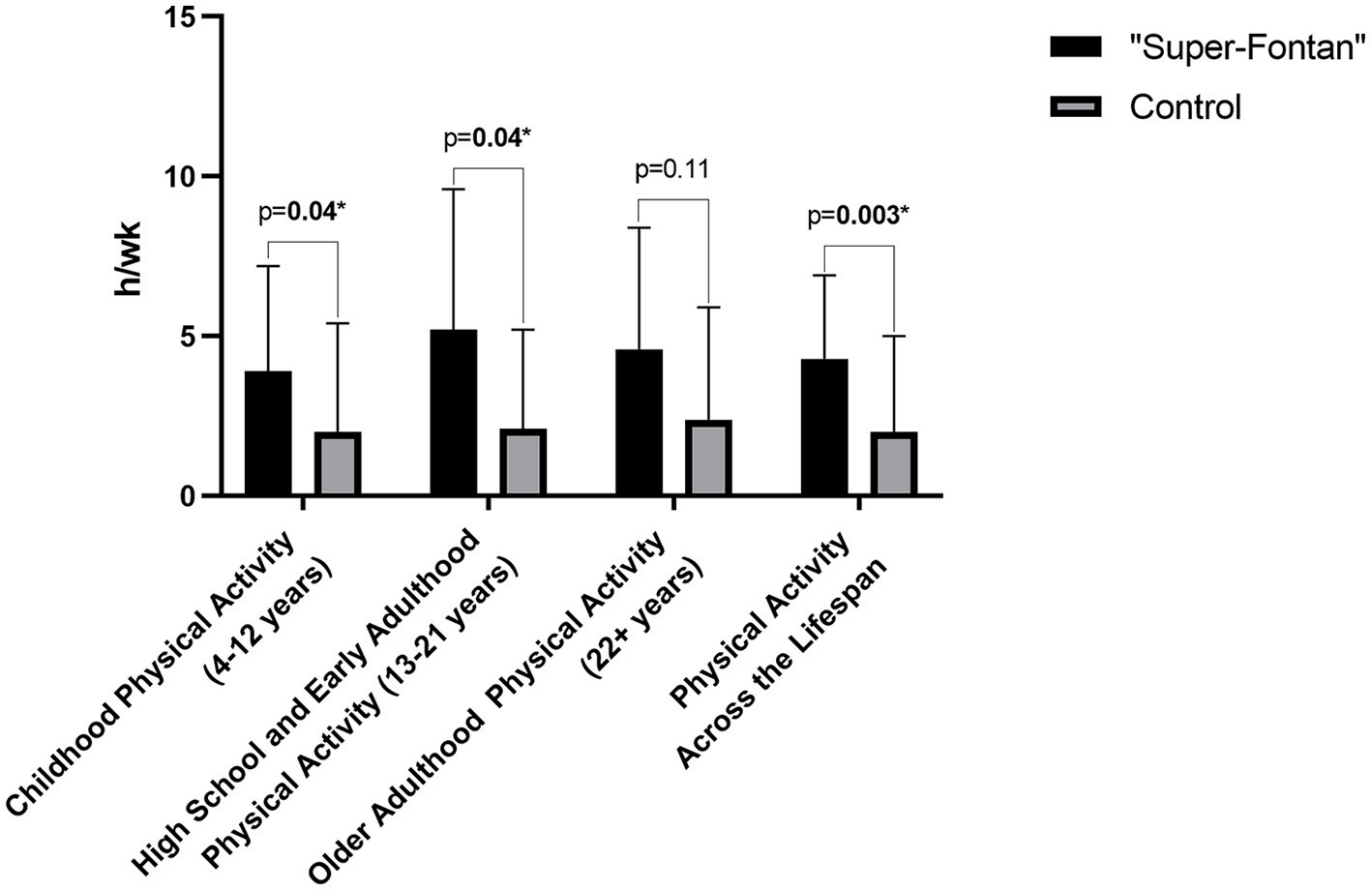
Hours per week (h/wk) spent participating in sports and physical activity.

One of the participants in the control group had an incomplete questionnaire and was excluded from the analysis. Results for the Kriska physical activity questionnaire are shown in Table 4; Figure 1. Childhood physical activity was higher in the “Super-Fontan” group compared to the control group (3.9 ± 3.3 h/wk vs. 2.0 ± 3.4 h/wk, *p* = 0.04). The average h/wk of sports and physical activity participation during high school and early adulthood was 5.2 ± 4.4 h/wk in the “Super-Fontan” group and 2.1 ± 3.1 h/wk in the control group, *p* = 0.04. There was no statistically significant difference in sport and physical activity participation during older adulthood. The overall average duration of sport and physical activity participation indexed per week was higher in the “Super-Fontan” group compared to the control group (4.3 ± 2.6 h/wk vs. 2.0 ± 3.0 h/wk, *p* = 0.003).

**Table 4:** The international physical activity questionnaire (IPAQ) and modified Kirska questionnaire results.

	**“Super-Fontan”**		**Control**		***p*-value**
	***n***		***n***		
**IPAQ**					
Work, MET-min/wk	10	2,002 ± 2,399	13	372 ± 889	0.06
Transport, MET-min/wk	10	306 ± 488	13	592 ± 721	0.10
Domestic and garden, MET-min/wk	10	1,519 ± 1,285	13	999 ± 1,483	0.23
Leisure time, MET-min/wk	10	1,214 ± 1,456	13	966 ± 1,141	0.83
Walking, MET-min/wk	10	2,015 ± 2,320	13	1,161 ± 1,419	0.78
Moderate activity, MET-min/wk	10	2,393 ± 1,794	13	1,294 ± 1,485	0.13
Vigorous activity, MET-min/wk	10	632 ± 584	13	474 ± 1,043	0.21
Total physical activity score, MET-min/wk	10	5,040 ± 2,209	13	2,929 ± 2,186	0.10
Sitting time, min/day	10	308 ± 123	13	453 ± 175	0.07
**Modified Kriska physical activity questionnaire**					
Childhood activity (ages 4–12), h/wk	10	3.9 ± 3.3	12	2.0 ± 3.4	**0.04**
High school and early adulthood (ages 13–21), h/wk	10	5.2 ± 4.4	12	2.1 ± 3.1	**0.04**
Older adulthood (22+), h/wk	10	4.6 ± 3.8	10	2.4 ± 3.5	0.11
Physical activity across the lifespan, h/wk	10	4.3 ± 2.6	12	2.0 ± 3.0	**0.003**

*Hours per week, h/wk; Metabolic minutes per week, MET-min/wk; Minutes per day, min/day. Bold values denote statistical significance (p < 0.05)*.

### Current Physical Activity Levels

**Figure 2:**
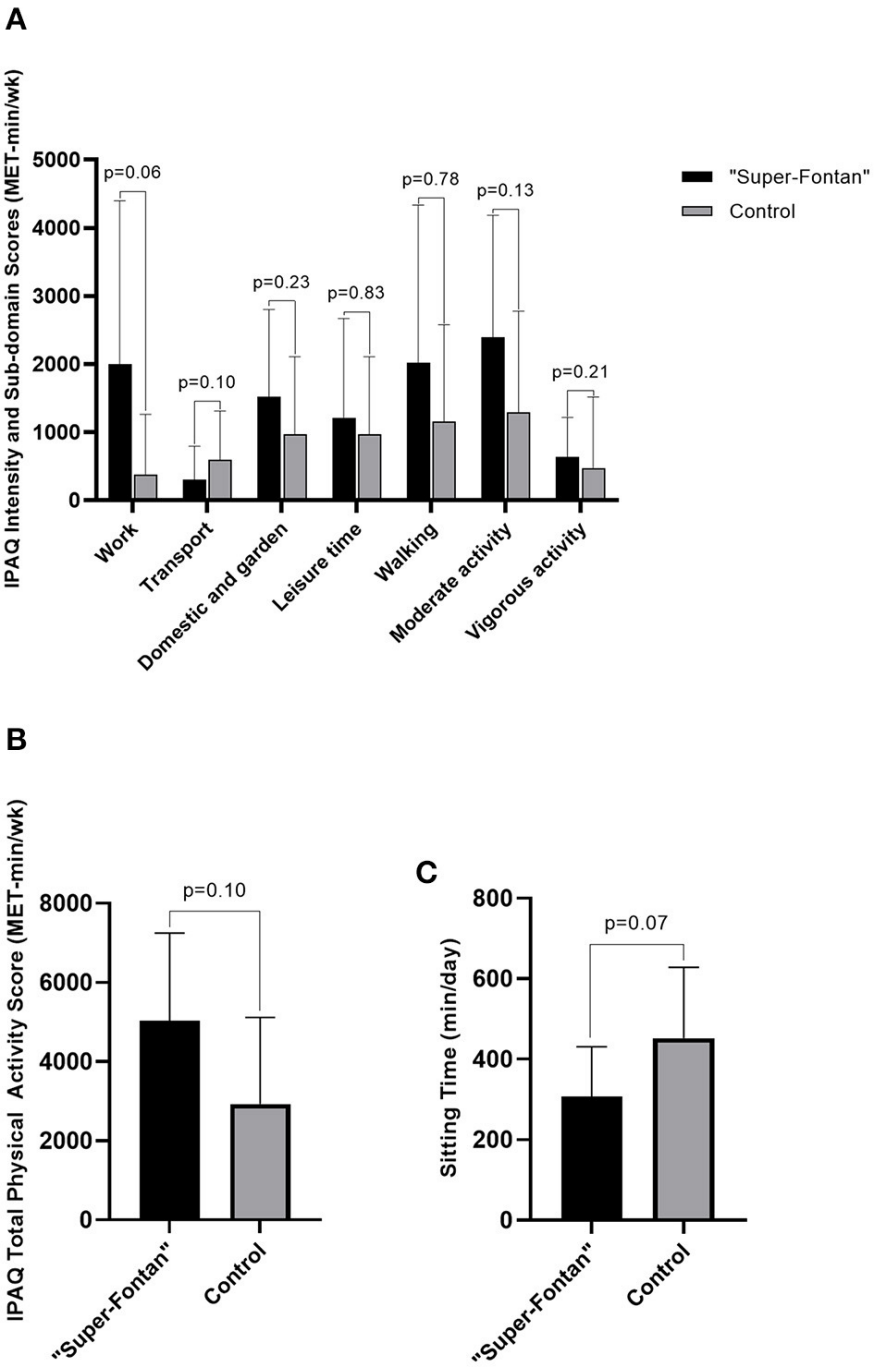
International physical activity questionnaire (IPAQ) scores. **(A)** IPAQ intensity and sub-domain scores, **(B)** IPAQ total physical activity score, **(C)** Sitting time. Metabolic minutes per week (MET-min/wk); minutes per day (min/day).

The detailed IPAQ results are shown in Table 4. The average self-reported MET-min/wk was higher at all physical activity intensities and sub-domains, except for the transport domain in the “Super-Fontan” group, although this did not achieve statistical significance (Figure 2). Sitting time tended to be lower in the “Super-Fontan” group compared to the control group (308 ± 123 vs. 453 ± 175 min/day, *p* = 0.07).

## Discussion

Despite an absent subpulmonary ventricle in the Fontan circulation, a subset of the population (“Super-Fontans”) can still achieve normal exercise performance, which is associated with increased levels of physical activity early in life, a healthy weight, and earlier age at Fontan completion.

### Factors Associated With Superior Physical Performance

In this study, the age at Fontan completion was lower in those with a “Super-Fontan” phenotype, and the absence of obesity or a dominant right ventricle were associated with normal exercise performance. This contrasts with previous findings that showed no differences between exercise performance Fontan phenotypes with these factors (6, 7). Our study describes an older Fontan cohort compared to previous series, and the conflicting findings might be attributed to an era effect. Alternatively, later age at Fontan completion, dominant right ventricular morphology, and obesity may manifest as important factors that impair the ability to achieve “normal” exercise performance later in life when circulatory function is more susceptible to compromise and maladaptation.

Similar to our findings, a review by Daley et al. found that earlier age at Fontan completion is associated with preserved long-term exercise capacity (25). A large multi-center series in a contemporary Fontan cohort also corroborates this; each year Fontan completion was delayed, percent predicted VO_2_ and HRR decreased by 1.5 percentage points and 4.1 beats/min, respectively (26). This association may be explained by enhanced reversal of adverse cardiac remodeling and offsetting volume overload with earlier age at Fontan completion (27, 28). Indeed, patients with a later age at Fontan completion present with evidence of greater ventricular dysfunction and atrioventricular valve insufficiency (29). Conversely, one study found a positive correlation between age at Fontan completion, and percent predicted peak VO_2_ (30). Although later age at Fontan completion is accompanied by an extended period of volume overload and cyanosis, it may allow for pulmonary artery catch-up growth and a larger conduit to optimize flow (31, 32); however, this theory requires verification.

We have previously reported increased adiposity is associated with a higher risk of adverse outcomes in people with a Fontan circulation (33). None of the people with the “Super-Fontan” phenotype were obese based on BMI compared to 22% in the comparator group with impaired exercise performance. The latest Pediatric Heart Network results also support this finding (34). This may be related to the impact of excess adiposity on respiratory muscle pump function and increased mechanical loading, which may impair exercise performance. In addition, the importance of maintaining a healthy body weight to preserve low pulmonary vascular resistance is increasingly recognized (32). Importantly, higher BMI often co-exists with increased visceral adiposity (epicardial and intra-abdominal fat), which may be particularly pathological—visceral adiposity is positively associated with pulmonary vascular resistance and inversely associated with ejection fraction and cardiac index in the Fontan circulation (35, 36). Of course, BMI may not be a robust measure of adiposity in the setting of complex congenital heart disease, where myopenia is common (33, 37, 38). The association between adiposity and pulmonary vascular resistance may be attributed to the adverse effects of pro-inflammatory adipokines (39), co-existing obstructive sleep apnea (40, 41), or decreased adiponectin (39, 42). The mechanisms underlying the association between adiposity and Fontan hemodynamics warrant further investigation.

We showed that an absence of a dominant right ventricle was associated with the “Super-Fontan” phenotype. A linear association between ventricular morphology and exercise capacity has previously been reported (43, 44). However, when categorized into exercise performance phenotypes (i.e., normal exercise performance vs. impaired exercise performance), series from Cincinnati Children's Hospital and the Children's Hospital in Philadelphia (a younger population than ours) found no association between ventricular morphology and a superior exercise performance phenotype (6, 7). It would seem plausible that a systemic right ventricle (compared to a systemic left ventricle) would be less likely to adapt to progressive hemodynamic perturbations over time and become more susceptible to circulatory demise and exercise intolerance. Long-term follow-up studies show dominant right ventricular morphology to be associated with worse clinical outcomes (45, 46).

We did not find any differences with regard to type of Fontan circulation, fenestration patency, or sex between the “Super-Fontan” phenotype and those with impaired exercise performance. However, although not statistically significant, there was a higher percentage of females in the “Super-Fontan” group compared to those with impaired exercise performance. Previous studies have also shown that a higher proportion of females are able to achieve better exercise performance (6, 7, 34), and have superior long term-outcomes (47, 48). The mechanisms underlying these sex differences are unclear and warrant further investigation.

Of note, while not statistically significant, and there were no differences in oxygen saturation, 20% of people with impaired exercise performance had a patent fenestration. It is unknown if this is associated with institutional bias toward fenestration or if these patients reflect a higher risk cohort requiring a fenestration at Fontan completion.

There were also no statistically significant differences in health-related quality of life measures between the “Super-Fontan” and control group in this study. However, the associations between quality of life and exercise performance are inconsistent across studies and warrant further investigation (49–51). This may be related to adults with a Fontan circulation accommodating to exercise intolerance over time. Supporting this notion, even asymptomatic people with congenital heart disease (New York Heart Association Functional Class I) have objectively impaired exercise capacity (2). Furthermore, the benefits of physical activity and exercise training on quality of life (particularly in the psychosocial domains) are potentially related to the social engagement that accompanies participation rather than better physical function.

### Exercise Response and Lung Function

HRR was greater in those with the “Super-Fontan” phenotype compared to the control group. Importantly, diminished HRR may be associated with arrhythmia-related mortality (9). This may explain why the combination of peak VO_2_ and HRR has a more substantial prognostic value for 5-year survival (52), with peak VO_2_ likely associated with heart-failure-related mortality. We also found higher peak circulatory power on average in people with the “Super-Fontan” phenotype, which is associated with better outcomes (53, 54). Collectively, the “Super-Fontan” exercise response reflects a low-risk Fontan phenotype.

Overall, most of our Fontan cohort had normal range chronotropic index implying an appropriate HR response relative to the metabolic load. Only three patients had a low chronotropic index with impaired exercise capacity indicating chronotropic insufficiency—It has been postulated that an inappropriate HR response is not a primary limiting factor to exercise performance unless severe chronotropic incompetence is present (55, 56). Exercise performance in the Fontan circulation appears predominately limited by preload deprivation that inhibits stroke volume augmentation. Over half of our Fontan patients with impaired peak VO_2_ show evidence of a cardiovascular (stroke volume) limitation to exercise performance denoted by an elevated chronotropic response or low peak oxygen pulse. Of course, the reduced oxygen pulse and elevated chronotropic index can also reflect impaired peripheral muscle oxygen extraction, which is also present in the Fontan circulation (57).

Lung function is commonly impaired in people with a Fontan circulation and is associated with prognosis and exercise capacity (58–60). The majority of our Fontan cohort with baseline spirometry results recorded had evidence of ventilatory restriction or mixed defect. Baseline spirometry abnormalities were less prevalent in the “Super-Fontan” phenotype compared to those with impaired exercise performance. However, despite abnormal lung function at rest, most patients in this study had ample breathing reserve, potentially because exercise performance in Fontan patients is predominantly impaired by cardiovascular limitations prior to encroaching upon ventilatory constraints. Only five people had evidence of mechanical ventilatory limitation during exercise, with no difference between the “Super-Fontan” and control groups. Of note, it is likely ventilatory limitations to exercise performance is underappreciated using breathing reserve in isolation as a surrogate. The addition of exercise flow-volume loops will perhaps reveal more Fontan patients with ventilatory limitations to exercise performance.

### Exercise Self-Efficacy and Physical Activity

Although self-reported physical activity levels were higher and sedentary (sitting) time was lower in the “Super-Fontan” group compared to the controls, this did not achieve statistical significance. This may be attributed to the duration between CPET and the administration of the questionnaires (~2 years), which likely does not truly reflect their current levels of physical activity. Furthermore, while the IPAQ shows moderate validity compared to accelerometers (61), in the setting of congenital heart disease, patients often overestimate their physical activity levels using the IPAQ long-form (62).

Indeed, Powell et al. reported that 77% of patients with the “Super-Fontan” phenotype participated in regular physical activity compared to 10% in those with impaired exercise performance (6). This is in accordance with our previous reports that show many of those with a “Super-Fontan” phenotype or positive exercise capacity trajectory regularly participate in moderate-to-vigorous sports and physical activity (5, 63). Importantly, increased physical activity levels could be attributed to higher exercise self-efficacy in the “Super-Fontan” cohort. Preceding studies have shown an association between physical activity levels and exercise self-efficacy (64).

We found that participation in regular structured sports and physical activity from childhood to early adulthood was significantly higher in those with a “Super-Fontan” phenotype compared to those with impaired exercise performance. Total overall participation in sports and physical activity was also higher in the “Super-Fontan” group. While exercise training interventions can increase peak VO_2_ (65, 66), it appears that participation in regular sports and physical activity from a younger age lays the foundation to achieve a low-risk “Super-Fontan” phenotype. This is consistent with the findings of Ohuchi et al. who showed that increased physical activity levels during childhood—reflected by a positive exercise capacity trajectory—were associated with better adult Fontan physiology, hepatic function, and prognosis (67). In another series, children and adolescents with a Fontan circulation who participated in sports during middle and high school had better lung function and exercise capacity (68). Regular sports, exercise training, or physical activity may be particularly crucial during childhood when organs, and especially the pulmonary vasculature, are likely more sensitive to adaptation in this period of rapid growth and development (67).

The association with regular moderate-to-vigorous physical activity participation and the “Super-Fontan”—superior exercise performance and low-risk—phenotypical expression may be attributed to multiple mechanisms. Regular participation in moderate-to-vigorous intensity sports and physical activity is important for the development of skeletal muscle mass and prevention of Fontan-associated myopenia (37). Deficiencies in skeletal muscle mass have important implications for ventricular function (37), and exercise capacity (57, 69). Higher appendicular muscle mass provides structural support to reduce venous compliance and enhances skeletal muscle pump function, facilitating preload and ventricular filling (70, 71). Indeed, increasing skeletal muscle mass through resistance exercise training improves ventricular filling, cardiac output, peak VO_2_, and reduces respiratory dependence in people with a Fontan circulation (72).

A primary constraint to ventricular filling appears to be elevated pulmonary vascular resistance. To maintain low pulmonary vascular resistance, there must be an adequately developed pulmonary circulation. However, pulmonary artery growth essentially ceases after Fontan completion (73), likely due to the combination of reduced pulsatility and pulmonary flow. We have previously shown that lower limb exercise can transiently increase (pulsatile) pulmonary flow (74). It is likely that engaging in regular long-term physical activity or exercise training (particularly during childhood), which transiently increases pulmonary flow (and may also alter the flow profile), facilitates pulmonary vascular development. Furthermore, periodic increases in ventricular filling associated with exercise may augment volume load to the chronically preload deprived ventricle and attenuate progressive “disuse hypofunction”. This phenomenon is observed when volume load is restored following atrial septal defect closure in adults, reversing diastolic dysfunction (31).

Of course, the association between the “Super-Fontan” phenotype and physical activity during childhood, and early adulthood, may simply reflect that those with superior exercise performance are more capable of participating in regular sports and physical activity, especially from an earlier age.

## Clinical Implications and Future Directions

While there appear to be common characteristics associated with the “Super-Fontan” phenotype, some patients in this subset still have features—such as a dominant right ventricle, atriopulmonary connection, or patent fenestration—that are expected to impede exercise performance. This suggests that extracardiac and potentially modifiable factors contribute to the expression of the “Super-Fontan” phenotype.

A key finding of our study is that exercise-self efficacy and regular participation in structured sports and physical activity from a young age is significantly higher in those with the “Super-Fontan” phenotype. This highlights the need to promote exercise training, sports, and physical activity in people with a Fontan circulation from early in life. Those who participated in sports from a young age probably also have higher exercise self-efficacy, which establishes a foundation for life-long physical activity habits. To date, exercise training is the most effective therapy for improving peak VO_2_ in people who have a Fontan circulation (70). While exercise training recommendations are now available for people with a Fontan circulation (65, 66, 75), they are predominantly based on clinical experience and expert opinion.

The forthcoming multi-center randomized controlled Fontan Fitness Intervention Trial (F-FIT) will hopefully provide more conclusive evidence to aid the development of future exercise training recommendations for people living with a Fontan circulation.

## Limitations

It is important to note that the retrospective design of this study can only show association and not causation. A key limitation in this study is the reliability of long-term physical activity questionnaires, which is subject to recall error. However, the reliability of long-term physical activity recall appears to be reasonable, with a previous study reporting an intraclass correlation coefficient of ~0.4 (76).

CPET is a specialized assessment that requires a high degree of clinical expertise to perform and interpret. This may restrict the results of this study to patients in the care of expert centers, which are predominately located in major cities. Indeed, we previously reported that <8% of people in the Australian and New Zealand Fontan Registry had recorded serial CPET documented (63). Therefore, our sample may be subjected to selection bias. The spirometry and CPET data were also derived from the tabulated reports available, and we had limited access to the flow-volume curves or primary CPET data. This restricted our ability to visually inspect the acceptability and repeatability of spirometry maneuvers, verify anaerobic threshold selection, or standardize the processing of CPET parameters. However, the spirometry and CPET studies were predominantly performed in “expert” experienced centers, and our results reflect the available reports used in clinical practice.

Our study is also limited in sample size, which increases the risk of a type II error, and we may not be able to detect some important associations.

Ideally, the function of the single ventricle and “Super-Fontan” status should be evaluated during upright exercise with invasive haemodynamic measures, but this is technically challenging. Currently, we and others have defined the “Super-Fontan” phenotype as achieving ≥80% predicted VO_2_ and/or work rate (5–7, 15), which can be influenced by the regression prediction equation selected and remains an arbitrary threshold.

## Conclusions

The “Super-Fontan” phenotype is associated with a healthy weight, younger age of Fontan completion (around 4 years), and higher overall levels of sport and physical activity participation during childhood and early adulthood. The “Super-Fontan” phenotype exercise response was accompanied by a higher HRR, oxygen pulse, peak circulatory power, and a later anaerobic threshold onset.

## Data Availability Statement

The original contributions presented in the study are included in the article/supplementary material, further inquiries can be directed to the corresponding author/s.

## Ethics Statement

The studies involving human participants were reviewed and approved by the Royal Children's Hospital Melbourne Human Research Ethics Committee. The patients/participants provided their written informed consent to participate in this study.

## Author Contributions

DT, Yd'U, DC, and RC contributed to the conception and design of the study. DT drafted the manuscript and acquired and analyzed the data. All authors critically reviewed the manuscript, contributed to the interpretation of the results, and approved the submission of the manuscript.

## Funding

DT was supported by a Grant from Additional Ventures, the National Heart Foundation of Australia Vanguard Grant (102277), the Medical Research Future Fund–Cardiovascular Health Mission–Congenital Heart Disease Grant (ARGCHDG000016), and the Paulette Isabel Jones PhD Completion Scholarship.

## Conflict of Interest

The authors declare that the research was conducted in the absence of any commercial or financial relationships that could be construed as a potential conflict of interest.

## Publisher's Note

All claims expressed in this article are solely those of the authors and do not necessarily represent those of their affiliated organizations, or those of the publisher, the editors and the reviewers. Any product that may be evaluated in this article, or claim that may be made by its manufacturer, is not guaranteed or endorsed by the publisher.
